# Removal of the Healthy Rather Than the Diseased Pulp

**Published:** 1917-05

**Authors:** I. N. Bromwell


### REMOVAL OF THE HEALTHY RATHER THAN THE DISEASED PULP

BY I. N. BROMWELL, D.D.S.

Primarily we recognize two chief functions belonging to the dental pulp, a creative function and a sustaining or nutritive function. We know that the former is absolutely essential up to an indefinite period, but so far as nutrition is concerned it has never been definitely shown that the presence of a vital pulp in a mature tooth is necessary for its preservation and continued functional worth. Notwithstanding the fact that positive evidence is wanting, there are many who believe that a pulpless tooth is not so good as one in which this structure remains intact. Personally I am of the opinion that a pulpless tooth is better than a tooth with a pulp, *unless that pulp is normal,* and I purpose to point out why I believe this to be true.

In the first place, the term "pulpless tooth" has been used in preference to a devitalized or dead tooth, because if the latter were the real condition, the tooth would be thrown off just as promptly as would any other foreign or inert body. But this does not occur, and we can all agree, therefore, on this one point, that a pulpless tooth receives some nourishment through the pericemental tissue, the cementum its normal amount and the dentin perhaps a slight amount through some osmotic process.

A pulpless tooth, after maturity, is immune to several pathologic conditions, which, broadly speaking, come under the head of constructive diseases and destructive diseases.

The following constructive diseases can not occur in a pulpless tooth: Calcareous degeneration of the pulp and dentin fibrillæ; secondary formations of dentin; pulp nodules and obliteration of the dentinal tubuli by tubular calcification.

The following destructive diseases common to the pulp can not occur in a pulpless tooth: Active hyperenia, both arterial and venous; inflammation, or pulpitis; suppuration in the form of ulceration or abscess; sclerotic changes in the pulp substance, or chronic inflammation; hypertrophy or fibroid degeneration and fatty degeneration.

The dentin of a pulpless tooth is immune to those pathologic conditions usually classed as adventitious dentins, five varieties of which are recognized, each dependent chiefly upon the presence of the pulp for their etiology. Pathological pigmentation of the dentin and calcification of the dentin tubules can not occur in a pulpless tooth. Internal absorption of dentin in which the walls of the pulp cavity become excavated and irregular in contour can not occur in a pulpless tooth, and finally what is classed as senile dentin, although a somewhat indefinite condition, can not occur in a tooth from which the pulp has been removed in earlier life. While the presence of the pulp in a mature tooth has little or no influence over pathologic conditions in enamel, acquired lesions in this tissue, such as abrasion, attrition, erosion, absorption, etc., are perhaps much better tolerated by a pulpless tooth than by one in which the pulp is retained.

The cementum is perhaps the only tooth tissue that is not directly affected one way or the other by the removal of the pulp, except perhaps that it may become somewhat more highly organized in a pulpless tooth, or it may become pathologic through periapical infection. If we were to recapitulate and enumerate, we would perhaps find that out of the several diseased conditions to which the tissues of a tooth are subject, about four-fifths of them can be prevented by the removal of the pulp, and there might be added to the list at least a consideration of dental caries.

I am not aware that there are any records to show that pulpless teeth are less liable to be affected by caries, and I do not know that they are, but when it is taken into consideration that with the removal of the pulp the dentin becomes less highly organized, it opens up a question for argument at least, and I am inclined to believe that the carious process goes on with less rapidity in a pulpless tooth on account of the changes which have taken place in the tubular contents.

If the foregoing statements are facts, and I believe they are conceded to be, there is left but one class of important pathologic conditions to deal with after removal of the pulp; those which appear in the apical region, all of which seems to be an argument in favor of discriminate pulp removal.

Several writers recently, and notably Dr. Thornton before this society at a recent meeting, have quoted Black as follows: "A tooth from which the pulp has been removed seems never to again completely recover." Doubtless Dr. Black's deductions were drawn from experiments made on teeth the pulps of which were diseased before removal, and therefore carries no weight as an argument against the removal of healthy pulps.

The term "mature tooth" has been used, and to derive the full meaning out of the manner in which the expression has been employed, it will be well to determine, if possible, just when a tooth has reached this important period in its existence. If we accept literally the definition of the word "maturity," it would correctly apply to the teeth only in very rare instances, for "the quality or period of complete growth or full development," as the definition reads, is seldom reached, the process of dentification and cementification being continuous from youth to old age under normal conditions. But the same might be said of other organs and structures in the body, so that in place of considering the subject from an anatomic, we are compelled to view it from a physiologic standpoint. Suppose, therefore, we fix upon the age thirty as the period when all teeth reach maturity, and we can logically do this, because by this time the dentin is present in sufficient bulk to fully serve its function and the pulp has reached the highest degree of efficiency. This latter statement is made somewhat guardedly, and only after considerable investigation along this line, in which it was found that in a very large percentage of cases the pulps in teeth over forty years old show a decided tendency toward a physiologic degeneracy or atrophy, and the older the tooth the more pronounced the degenerative changes. This fact may be demonstrated by the simple experiment of removing the pulps from recently extracted teeth of older persons, and examining the same with a magnifying glass, with the result that a large percentage will show evidence of degeneration; and under the microscope a great variety of abnormalities may be seen.

During the preparation of this paper, the writer broke up a number of freshly extracted teeth from persons ranging in age from forty-five to sixty-eight, and in a large percentage the pulps showed marked signs of degeneration.

Some of the more recent writers dealing with the subject under discussion appear to believe that complete death of the pulp is essential to pericemental and peridental disease and discomfort. I not only take exception to this, but wish to add that the pathologic conditions in tooth structure and pulp tissue, without complete death of the latter, are chiefly responsible for most of the obscure painful symptoms in and about the teeth, these conditions being very much more frequently met in vital teeth thus affected than in well-cared-for, pulpless teeth.

In this connection is it not well to differentiate between the treatment and filling of a root canal in which normal conditions were present in the pulp before removal, and the reverse class in which there may have been a variety of diseased conditions involving the dentin, the pulp and pericemental tissue? In the former case it is not a question of "treatment," it is purely a surgical operation, recovery from which should be prompt and permanent; in the latter it is a case of "treatment," and *all* of the diseased parts must be made well before recovery can be expected.

It is difficult to believe if a normal tooth is isolated by the rubber dam, its pulp removed, the canal sterilized, dehydrated, thoroughly filled and hermetically sealed, that there is a possibility of infection from an extrinsic source thereafter.

But how about the tooth that has passed through one or more of the pathologic stages, encouraged and actually produced in the pulp by the irritating effect of a shell crown or large metallic filling or inlay? This is a far different story. In these cases the seeds of infection have already been sown, lodging themselves in the dentin tubuli or perhaps in the more congenial tissue which occupies the apical space. We must not therefore lose sight of the fact that periapical infection and inflammation is possible from another or intrinsic source, and that the normality or abnormality of the tooth will have a decidedly controlling influence over this.

Pathologists are of the same opinion on at least one point, that is, the possibility of infection in any part of the body reached by a capillary blood supply. It is a well-known fact that pyogenic germs may be present in the blood without local disturbance, and this may continue for a long time, but with a local irritation in the nature of an injury the germs may be held up, passing from the capillaries to the surrounding connective tissues, promptly followed by inflammation and suppuration. While a small percentage of apical abscesses may be formed in this way, it is not logical to believe that their appearance is less likely after the careful removal of healthy pulps than after the removal of pulps pathologically affected.

In my previous paper, the simple facts were stated that certain degenerative changes took place in the pulp under certain irritating influences, but no reference was made to the more or less intricate process through which this took place. The fact has been established that the pulp of a normal tooth will tolerate a variation of temperature from 65 degrees to 105 degrees F., and that even extreme thermal changes above or below these points, while frequently causing pain, will be tolerated if not too long indulged in. But with the removal of a portion or all of the enamel, thus exposing the dentin or decreasing the thickness of dentin covering the pulp, there results a marked change in the disposition for the pulp to respond to thermal stimuli, be it either above or below the specified range of tolerance. That is, the reaction to thermal stimuli is promptly increased, and the irritating effect becomes more and more pronounced. If, therefore, we add the exciting influence of a gold cap, or a large metallic inlay, it is plainly evident that with the increased thermal activity the tendency toward shock to the pulp is materially increased.

I have already referred to the fact that the exposure of dentin to the slightest degree, either by mechanical means or through pathology, opens the way for pulp irritation, and to this might be added, "the greater the exposure of dentin the greater the chances for irritation." Also, the *rapidity* with which this exposure of dentin is brought about has much to do with the results which follow. *Freshly cut cavities for inlays to be used as abutments for bridges will result in much more pulp irritation than those created by slow process of caries. Likewise the hasty removal of enamel and resultant instantaneous exposure of dentin will be followed by greater pulp irritation than the slow process of enamel destruction in cases of mechanical or chemical abrasion. The former in each instance prohibits the possibility of nature's help in protecting the delicate pulp structure, while the latter in each instance encourages this.*

Perhaps the first influence to be felt by the pulp under such conditions is one which shows in the form of one or more of the constructive diseases to which the pulp is subject, these including calcareous degeneration of the pulp, secondary dentin, formation of pulp nodules, calcification of the dentin tubules, etc. If the influence is one resulting in calcic degeneration, we have a condition quite similar to pathologic calcic degeneration in other parts of the body. In other words, there is a permeation or infiltration of inorganic material into the substance of the pulp from the lymph, a process which gradually results in degeneration and possible eventual death of the tissue.

Susceptibility may be a very important factor in this disease of the pulp, but nevertheless without the existing cause, the gold cap or the large inlay, the process of calcic degeneration is not so likely to occur. During the active stages of progressive calcic degeneration the pulp retains its vitality, but the pathology asserts itself in a gradual but persistent change in the character of the pulp substance through a destruction of its cellular elements; a change so marked that it may be readily recognized by the naked eye in an extracted pulp.

If by some possible system we could within a brief period induce in the pulp substance a condition similar to that brought about through calcic degeneration, many of the difficulties of pulp removal would be overcome, because we find in these cases little or no resistance to removal through odontoblastic attachment to the canal walls, the leather-like granular condition of the pulp permitting its removal *en masse*, even the smallest hairlike processes coming away without fracture.

Calcic degeneration of the pulp can be suspected when there is obscure reflex pain, when the response to thermal changes is delayed, but finally responds to slightly elevated temperature. Such symptoms as these call for an X-ray examination which may or may not materially assist in diagnosis.

Calcification of the dentin tubules might be considered as a disease of the dentin, but it is usually classified with the diseases of the pulp, owing to the intimate relationship between the pulp and the tubular contents. Perhaps no other constructive disease of the pulp is more readily brought about by mechanicothermal influence than this of tubular calcification, because it is the continued and prolonged mild irritation such as is most apt to follow the placing of shell crown or inlay that is responsible for this pathology. Here through an atrophy of the fibrillæ, and a reduction in the size of the lumen of the tubules, we have practically a condition of dentinosclerosis, coincident with which there is a change in color, a kind of translucence in the tissue, with lessened sensibility.

By some it is argued that this is a physiologic process to protect the pulp, and to a certain extent and for an indefinite period, this is correct, and while the disintegration of the pulp is delayed for a time, this will eventually follow.

Secondary dentin, while usually classed with constructive diseases of the pulp, might as well be included in the destructive diseases of this organ, knowing as we do that if the process is kept up long enough, it would mean the obliteration of the pulp cavity as well as its contents.

I believe I am justified in making the statement that in every case of shell crown or large metallic inlay, we will have an abnormal deposit of dentin in the wall of the pulp cavity. As to the real physiologic value of this newly and rather hastily formed layer of dentin little appears to be positively known; however, I believe that were it possible to make some arrangement with nature to do our root canal filling by this method, it would be a marked improvement over guttapercha, chloropercha and other inorganic substance now more or less unskillfully and, therefore, unsatisfactorily employed. The real damage to the pulp through a deposit of secondary dentin, does not lie in the deposit itself, but it is the result of overstimulation and over-activity of the pulp cells and its vascular supply. It has been repeatedly argued that a definite layer of cement between the shell crown or inlay and the dentin of the tooth will protect the pulp from shock. As a matter of fact, the average oxyphosphate cement will in an indefinite period result in about as much pulp disturbance as the metals themselves, and in no instance can cement thus interposed be depended upon to permanently protect the pulp if it comes in direct contact with the dentin.

The pulps of the teeth that have been ground down and covered with porcelain jacket crowns are susceptible to the same irritating influences and final results, the only difference being that the thermal activity is not so pronounced, and the destructive changes take place somewhat more slowly.

Pulp nodules, while frequently found in apparently healthy teeth, that is, teeth without caries and free from irritating mechanical influences, are much more likely to occur and to grow with greater rapidity and to greater proportions in teeth, the pulps of which are under the influence of some external irritant. There is some difference in the character of these growths. In the majority of instances pulp nodules in otherwise healthy teeth are most apt to be the floating or unattached variety, while in those cases in which the pulp is under the influence of some surface irritant, the nodular growths are likely to be attached to the walls of the pulp chamber. I believe it is a well understood fact that only in a small proportion of cases are the unattached nodules a cause of pain, and this is especially true if they occupy the central portion of the pulp chamber. The reason for this is explained by the statement that they represent the actual calcification of just so much pulp substance, and as such, cause little or no pressure on the surrounding parts. On the other hand, the nodular deposits attached to the pulp chamber wall are more likely to cause pain because of their close association and possible pressure upon the capillary loops and nerve terminals so much in evidence in this part of the pulp. In the former case, the floating pulp stone is not an odontoblastic product, while in the latter it is very likely to be. The foregoing is, therefore, another argument in favor of discriminate pulp removal when the crown of a tooth is to have added to it a mechanically irritating substance.

Of the destructive diseases affecting the pulp through the influence of thermal stimuli, arterial hyperemia is, perhaps, the most common. While this condition may occur and does occur as a result of the slow destruction of tooth substance by erosion, abrasion, caries, etc., in these instances there is an opportunity for the pulp to protect itself and active hyperemia is, in many instances, delayed indefinitely. But not so when the enamel and, perhaps, a part of the dentin is mechanically removed for shell or jacket crowns or large inlays. The fibrillæ are not fortified against such an unexpected onslaught, and, as a result, the blood vessels of the pulp become dilated coincidently with the attachment of the crown or inlay, and while there may be no symptoms of hyperemia sufficient to bring a complaint from the patient, the disease is present, as the first in the chain of destructive pulp conditions. There is only one rational condition for this treatment, that is, the removal of the irritating cause, and this to the conscientious operator is an embarrassing procedure, to say the least, after having completed and inserted an otherwise satisfactory piece of work. It is scarcely necessary for me to relate in detail the pulp pathology that is sure to follow this primary irritation: acute inflammation, sclerosis, fibrid degeneration, etc. Right here is another opportunity to insert the thought uppermost in this paper: *remove the pulp before it becomes diseased.*

It is not to be supposed that any of the destructive diseases of the pulp are to be confined and limited to the pulp tissue alone. Many of them and, in fact, all of them extend beyond the walls of the pulp cavity, first into the tubuli, and then into the more susceptible tissue occupying the so-called apical space, and in this way local, and perhaps systemic and organic infections are established.

While this paper has for its chief object the advocacy of the removal of healthy pulps whenever and before there is a possibility for them to become diseased, thus inviting a multitude of complications both local and general, I am not quite ready to accept the views of certain extremists in both the medical and dental professions who pretend to believe that nearly all inflammatory conditions in organs and tissues, far remote from the teeth, may have their origin traced to disease about the root apices. While I believe it is possible for an aggravated periapical infection of long standing to result in remote organic and structural changes of a very serious nature, I do not believe this to be nearly so common as some writers would have us understand; instead I am more inclined to believe that the periapical disease is just about as often the result of some systemic or organic deflection localizing itself, by the passage of pyogenic germs from the capillaries into the already depressed connective tissue.

While it is not my intention nor is it expedient to go into the technic of pulp removal and canal filling, I will briefly touch upon one phase of the subject seldom taken into consideration. In the majority of instances the operator appears to be content with the removal of the bulky pulp substance, or what may be considered the pulp proper. Especially is this so if the pulp comes away *en masse*. While the extraction of the pulp in this manner brings with it the odontoblastic layer and possibly a portion of the processes penetrating the dentinal tubuli, it leaves behind that layer of indifferent tissue technically known as the dentinogenetic zone. This layer of indifferent tissue is highly organized and is made up of a proportionately greater thickness in young teeth than in those more mature, and its removal by reaming is highly essential before filling the canal in all cases, and especially when pulp disease has previously existed.

In a careful study of periapical infection and inflammation, some consideration should be given to the nature of the connective tissue occupying the apical space. Authorities differ as to the true histologic nature of the alveolodental membrane in general, and this difference of opinion applies with equal force, and, perhaps, has a special significance when considering the membrane immediately surrounding the root apex. It will be noticed that the term "alveolodental" is used in preference to "peridental" or "pericemental" membrane. The chief difference of opinion as to the histology of the membrane lies in the question of its duality, and I am one of those who believe the membrane to be of a dual nature, and thus the preference for the term "alveolodental." A careful microscopic examination of this membrane shows two chief varieties of cells, cementoblasts and osteoblasts, the former occupying the dental side of the tissue and the latter the alveolar side. In addition to these cells, we have also the supporting fibres which pass from bone to cementum, the whole making up a highly organized connective tissue. The chief blood vessels and nerves of the membrane pass between the two layers of constructive cells, and seem in a measure to separate the dental portion of the membrane from the alveolar portion. While in a measure the cementoblasts and the osteoblasts primarily have a somewhat similar function, it must be remembered that ultimately they become as different as life is from death, and this statement really explains the difference. The osteoblasts continue after becoming encapsuled in the bone as live cells, the lacuni and canaliculi, while the cementoblasts, after becoming encapsuled in the cementum, become the inert cement corpuscles and do not have the power of nutrition or reconstruction. It would, therefore, appear that the question of the successful treatment of periapical disease would largely depend upon the structure or structures affected, those cases involving only the alveolar portion of the membrane together with the adjacent alveolus, responding, through physiologic help, much more quickly than those involving the dental portion with its adjacent cementum. In other words, the reconstruction of bone, or, perhaps, a better term—the repair of bone, is a comparatively simple process, while the reconstruction of cementum is improbable, and its repair is a complicated and uncertain process.

Dental literature coming out of New York City and its environments has recently contained much about a newly coined term in dental pathology, a "granuloma." While I do not pose as an authority on general pathology (in fact, I am quite willing to admit that what I do *not* know about it would make quite a book), I have, through careful reading and through personal contact with authorities on the subject, gained information which prompts me to close my paper with an unfavorable criticism of the use of this word as now applied to the repair of periapical tissue.

A granuloma, so-called, can only be present as a secondary process, never as a primary one. Such growths are brought about in the following manner: The irregular openings which remain in the tissue after the completion of an abscess evacuation must in some way be repaired. The invading leukocytes gradually become freed from the abscess walls, and the necrotic tissue which forms the pyogenic membrane gradually becomes covered with groups of growing connective tissue cells known as granulation tissue, and when this assumes a tumor-like mass, as it frequently does in tubercular and syphilitic patients, then forms a granuloma.

Authorities appear to differ as to the application of the term "granuloma," as now so frequently applied to periapical disease, but of one thing there can be no mistake; that is, unless there has been an active inflammation followed by suppuration, in other words, what we commonly call an alveolar abscess, there can be no granuloma, because the existence of the former is essential to the establishment of the latter. From the fact, however, that in the process of repair following an apical abscess, there is no neoplastic activity, nothing definite in the nature of a tumor, it would seem that the term "granuloma" is a misnomer.—Reprinted from *Items of Interest.*

